# Specific Reaction Parameter Multigrid POTFIT (SRP-MGPF): Automatic Generation of Sum-of-Products Form Potential Energy Surfaces for Quantum Dynamical Calculations

**DOI:** 10.3389/fchem.2019.00576

**Published:** 2019-08-14

**Authors:** Ramón L. Panadés-Barrueta, Emilio Martínez-Núñez, Daniel Peláez

**Affiliations:** ^1^Laboratoire de Physique des Lasers, Atomes et Molécules (PhLAM), Université de Lille, Villeneuve-d'Ascq, France; ^2^Departamento de Química Física, Facultade de Química, Universidade de Santiago de Compostela, Santiago de Compostela, Spain

**Keywords:** PES, sums-of-products, tensor-decomposition, quantum dynamics, reparametrized semiempirical, TSSCDS, global optimization

## Abstract

We present Specific Reaction Parameter Multigrid POTFIT (SRP-MGPF), an automated methodology for the generation of global potential energy surfaces (PES), molecular properties surfaces, e.g., dipole, polarizabilities, etc. using a single random geometry as input. The SRP-MGPF workflow integrates: (i) a fully automated procedure for the global topographical characterization of a (intermolecular) PES based on the Transition State Search Using Chemical Dynamical Simulations (TSSCDS) family of methods;i (ii) the global optimization of the parameters of a semiempirical Hamiltonian in order to reproduce a given level of electronic structure theory; and (iii) a tensor decomposition algorithm which turns the resulting SRP-PES into sum of products (Tucker) form with the Multigrid POTFIT algorithm. The latter is necessary for quantum dynamical studies within the Multiconfiguration Time-Dependent Hartree (MCTDH) quantum dynamics method. To demonstrate our approach, we have applied our methodology to the *cis-trans* isomerization reaction in HONO in full dimensionality (6D). The resulting SRP-PES has been validated through the computation of classical on-the-fly dynamical calculations as well as calculations of the lowest vibrational eigenstates of HONO as well as high-energy wavepacket propagations.

## 1. Introduction

A detailed knowledge of the topography of a Potential Energy Surface (PES) is a highly desirable prerequisite for the simulation of any dynamical process. Topography on its own, however, does not fully determine the behavior of a system and dynamics calculations become mandatory (Tuckerman et al., 2002; Peláez et al., 2014). Furthermore, for an accurate theoretical description of molecular processes (spectroscopy, reactivity), one should, if possible, resort to nuclear quantum dynamics calculations (Gatti, 2014). In the specific case of vibrational problems, powerful methods based on the resolution of the time-independent Schrödinger equation exist such as vibrational self-consistent field/vibrational configuration interaction (VSCF/VCI) (Rauhut, 2007; Neff and Rauhut, 2009), vibrational second-order perturbation theory (VPT2) (Barone, 2005) and vibrational coupled-cluster theory (Christiansen, 2004). For an extensive and recent review of some of them, the reader is referred to a recent publication (Puzzarini et al., 2019). However, owing to our interest in describing chemical processes, we shall turn our attention toward methods able to describe wave packet propagations. In this context, within the last few years, we have experienced a boost in dynamical methodologies capable of describing the dynamics of molecular systems up to medium-large size, ranging from semiclassical (Levine et al., 2008; Shalashilin, 2010) to fully quantal (Gatti, 2014). With respect to the latter, by far, the most popular approaches nowadays are those based on, or related to, the grid-based Multiconfiguration Time-Dependent Hartree (MCTDH) algorithm (Beck et al., 2000). In MCTDH, a molecular wavefunction (WF) is expanded in a basis of time-dependent nuclear orbitals. Taken MCTDH as reference, two powerful multiconfigurational methods exist. On the one hand, the partially grid-based G-MCTDH method in which some of the time-dependent basis functions are substituted by (typically frozen) Gaussians functions (G) (Burghardt et al., 2008), and the Variational Multiconfigurational Gaussian (vMCG) method (Richings et al., 2015) (and its direct-dynamics (DD) extension) which are grid-free and use Gaussian functions only. For the sake of completeness, one should mention the recent and promising direct-dynamics approach of MCTDH by Richings and Habershon (2018).

It should be evident that the quality of the results of any dynamical calculation is limited by the accuracy and efficiency of the underlying electronic structure method used to represent the PES, either globally (as in grid-based methods) or locally (on-the-fly approaches). When expressed *globally* on a grid, formally as a multidimensional tensor, the limitation lies on the number of dynamical degrees of freedom and the possibility of fitting the PES to an appropriate functional form. In the case of on-the-fly methods, on the other hand, the number of degrees of freedom (DOF) it is not the main limiting factor but the *number of electrons*, in other words, the level of theory and its performance in the form of electronic structure software *calls* (energies, gradients, Hessians) at each time-step. This fact constrains on-the-fly approaches to modest levels of theory.

Obtaining a fit for a high-dimensional PES is a complex and tedious task. Whatever the approach, any fitting procedure requires a more or less large set of reference values (molecular energies and/or gradients and, possibly, properties such as dipoles) which will constitute the data to which an algorithm will try to fit a given function. *Ad hoc* analytical functions are usually added to the resulting fit in order to ensure a correct physical behavior, for instance in the asymptotic regions, or to guarantee a correct periodicity of the potential as in the case of rotors. Focusing on the fitting methods typically used in combination with nuclear quantum dynamical approaches, many techniques have been proposed. To name but a few, popular methods include the permutationally invariant polynomials (Braams and Bowman, 2009), the interpolating moving least-squares (Dawes et al., 2007), the triatomics-in-molecules approximation (Sanz-Sanz et al., 2013), Shephard interpolation schemes (Frankcombe and Collins, 2011). Moreover, for more than a decade now, Neural Network (NN) approaches have (re)gained preeminence being triggered by the pioneering work of Manzhos and Carrington (2006) and, very recently, their application to MCTDH by Pradhan and Brown (2017). In this line, Jiang and Guo have gone a step further and have developed a NN approach with implicit nuclear permutational symmetry (Jiang and Guo, 2014). For the sake of completeness, one should mention the works of Rauhut (2004) and Sparta et al. (2010) in which PESs for vibrational calculations are generated in an automated and adaptive fashion. Powerful and accurate as these methods are, a high degree of expertise is still required to master and to apply these techniques, particularly for medium-large systems (≥6D), thus preventing them from a wider-spread use. Furthermore, in studies where external fields (e.g., a laser) are needed, surfaces of molecular properties are also required and, as a consequence, extra fits are necessary.

In this work, we present Specific Reaction Parameter Multigrid POTFIT (SRP-MGPF), a method which provides a well-balanced solution to the aforementioned issues. SRP-MGPF is able to generate a chemically-accurate PES as well as the same-level-of-theory molecular properties surfaces, starting from a single input geometry and requiring minimal intervention of the user. In this sense, we can safely affirm that the procedure is *quasi* black-box in nature. SRP-MGPF relies on three main steps: (i) generation of a set of reference geometries (energies and properties); (ii) reparametrization of a semiempirical Hamiltonian (Specific Reaction Parameter Hamiltonian, SRP) based on the previous information; and (iii) tensor-decomposing the SRP with MGPF. We shall focus on the *standard* MCTDH method for which a global PES needs to be fitted into some kind of functional form and, typically, refitted to a grid. Furthermore, our results can also be directly applied to any on-the-fly methodology. It should be highlighted at this point that reparametrized semiempirical Hamiltonians have been typically used in direct dynamics studies as well as in kinetic studies (Rossi and Truhlar, 1995; Troya, 2005; Rodríguez-Fernández et al., 2017). Moreover, semiempirical Hamiltonians have been successfully used in describing dynamics on electronically excited states (Toniolo et al., 2003; Silva-Junior and Thiel, 2010). It should be stressed that SRP methods qualify as quantum chemical ones. As such an SRP does not include, necessarily, any fitting functions. Hence, the SRP parameters obtained through our fitting process will define a level of electronic structure close to a high-level reference one.

In our approach, as generator set for the reference fitting points, we employ the so-called Reaction Network (RXN) (Martínez-Núñez, 2015b), i.e., the *complete* set of stationary points (minima, transition states,…) of a PES. The RXN captures the main topographical (even topological) features of the target PES and thus constitutes a sensible choice for the reference set. Characterization of the topography of a PES is, however, not an evident task. To this end, we make use of the recently developed Transition State Search Using Chemical Dynamics Simulations (TSSCDS) (Martínez-Núñez, 2015a,b) method which relies on the efficient sampling of configuration space combined with a graph-theory based identification of transition state (TS) structures, which are finally optimized and the corresponding Minimum Energy Paths obtained with standard methods. The TSSCDS approach has been recently extended to specifically study van der Waals complexes (vdW) or, more generally, non-covalently bound systems (vdW-TSSCDS) (Kopec et al., 2019).

A set of *optimal* semiempirical Hamiltonian parameters is then obtained by global minimization of the Root-Mean Square Error (RMSE) between a set of reference *ab initio* energies, for instance, (on the RXN-derived geometries) and the corresponding SRP ones. The SRP approach to PESs presents interesting features that make it very appealing when compared to formally higher-level methods (Density Functional Theory, DFT, or *ab initio*). First, SRPs are fast-computing parametrized electronic structure methods, some of the integrals are neglected while the remaining are parametrized to reproduce high-level results. As such, they typically exhibit a *correct* physical behavior. Second, in contrast to other *fitting* procedures (for instance based on any kind of polynomial expansions or neural networks), SRPs exhibit a correct behavior outside the fitting boundaries, *if* the SRP parameters remain somewhat physical (*small* variation with respect to their reference values). Third, by varying the SRP parameters we can simultaneously fit both energies and the molecular properties accessible to the semiempirical software. It should be highlighted that in the usual approach energies and properties (e.g., dipole) are computed at a set of reference geometries and then need to be independently fitted to either potential energy surfaces or property surfaces (x-dipole, y-dipole, etc.). In contrast, in our method a single optimization process suffices to yield a simultaneous fit of all properties simultaneously, provided that information on the desired properties is included in the reference data. Last, but not least, the number of parameters is *independent* of the number of atoms. They only depend on the number of different atoms (and possibly on their chemical function) and, as such, it is in a sense not affected by the curse of dimensionality. In our specific approach, we have used as base model chemistry the Parametric Method 7 (PM7) method as implemented in the OpenMOPAC software package (Stewart, 2016). This choice is justified by the quality of the obtained results as well as its efficiency in terms of computational time (PM7 is orders of magnitude faster than *ab initio* methods) (Stewart, 2013).

The final step, specific for grid-based methods, is the tensor-decomposition of the SRP-PES into an appropriate form. To this end, we utilize the Multigrid POTFIT (MGPF) algorithm (Peláez and Meyer, 2013), succinctly described in section 2.3. MGPF has been successfully applied to the computation of vibrational eigenstates (Peláez et al., 2014), infrared (IR) spectra (Peláez and Meyer, 2017), and electron dynamics including continuum (Haller et al., 2019) With SRP-MGPF, owing to the extreme efficiency of the semiempirical calculations, we can directly generate the SRP-PES on a grid.

This manuscript is structured as follows. In section 2 we provide a succinct introduction to the methods employed in our workflow. In section 3, which presents the application of our novel methodology to the HONO molecule in full-dimensionality, we carefully discuss all specific aspects related to the actual calculations. Section 4 concludes the paper and gives some hints on future developments and possible applications of the method.

## 2. Theory and Computational Details

Our automated methodology for computing a global PES consists of three steps: (1) automatic and global determination of stationary points (minima and transition states), as well as the corresponding Intrinsic Reaction Coordinate paths (IRCs), the so-called Reaction Network (RXN); (2) reparametrization of a semiempirical Hamiltonian (SRP) to reproduce a desired level of electronic structure theory (e.g., *ab initio*) using the RXN and neighboring points; and (3) tensor-decomposition of the SRP Hamiltonian with the MGPF algorithm. It should be noted that after stage (2), we already have a global PES which can be used in conjunction with any type of *on-the-fly* dynamics scheme. We shall describe in the following each of the above mentioned stages. First of all, we shall discuss our specific procedure for the reparametrization of semiempirical Hamiltonians. Then, we shall present our way of generating a set of reference points based on the RXN obtained using the (vdW-)TSSCDS method (Martínez-Núñez, 2015a,b). Subsequently, we shall discuss how we integrate this information in combination with the NLOpt (Johnson, 2011) library and the openMOPAC software (Stewart, 2016) to produce an *optimal* set of SRP parameters. The resulting SRP-PES is then *interfaced* with MCTDH through the Multigrid POTFIT program (Peláez and Meyer, 2013) thus generating a SRP-MGPF PES on the grid and in sums-of-product (SOP) form.

Finally, it should be highlighted that, for the graphical representations, we have made extensive use of the SciPy scientific tools by Jones et al. (2001).

### 2.1. Global Optimization of Semi-empirical Hamiltonians Parameters

Semiempirical potentials can be seen as parametrized Hartree-Fock methods in which some of the electronic integrals are either neglected or replaced by parameters obtained as fitting constants using large sets of reference data (high-level *ab initio* calculations and/or experimental data) (Stewart, 2013; Thiel, 2014). In this sense, semiempirical methods lie somewhere between force fields and *ab initio* methods (Stewart, 2013). Owing to the lower amount of integral calculations, semiempirical methods are orders of magnitude faster than *ab initio* methods and, hence, they are routinely used in the study of large systems (Christensen et al., 2016). In addition to this, with a suitable configuration interaction formalism, semiempirical methods can also be used for the study of excited states (Toniolo et al., 2003; Silva-Junior and Thiel, 2010). A milestone in the usage of semiempiricals was achieved by Rossi and Truhlar (1995) who introduced the idea of reparametrizing a semiempirical Hamiltonian in order to reproduce a given high-level *ab initio* level of theory for a *specific* chemical reaction (or family thereof), hence the name of Specific Reaction Parameter (SRP) Hamiltonians. Since then, this technique has been successfully applied to the study of chemical reactions of large-dimensional systems using classical dynamics (Layfield et al., 2008) as well as to kinetic studies (Rodríguez-Fernández et al., 2017). In the present work, we go a step further and will use the SRP approach for the generation of a PES suitable for quantum dynamical studies. To this end, we used the publicly available non-linear global optimization library NLOpt (Johnson, 2011) to reparametrize the PM7 semiempirical model (Stewart, 2013) as implemented in openMOPAC (Stewart, 2016). The choice of PM7 responds not only to its proven accuracy but also to the fact that it includes diatomic parameters in addition to the standard atomic ones, thus providing extra flexibility to the optimization process (Stewart, 2013). Hereafter, we shall refer to the set of SRP parameters as ${\left\{\zeta_{i}\right\}}_{i=1}^{D}$, being *D* the total number of parameters. It is important to notice that the latter depends on the number of *atom types* and *not* on the dimensionality of the system. It should be stressed that we are dealing with a *fitting function* which has an implicit physical character (HF-like) and, as such, it is expected to yield a *global* qualitatively-correct behavior and to require less fitting points than other traditional fitting approaches.

**Figure 1:**
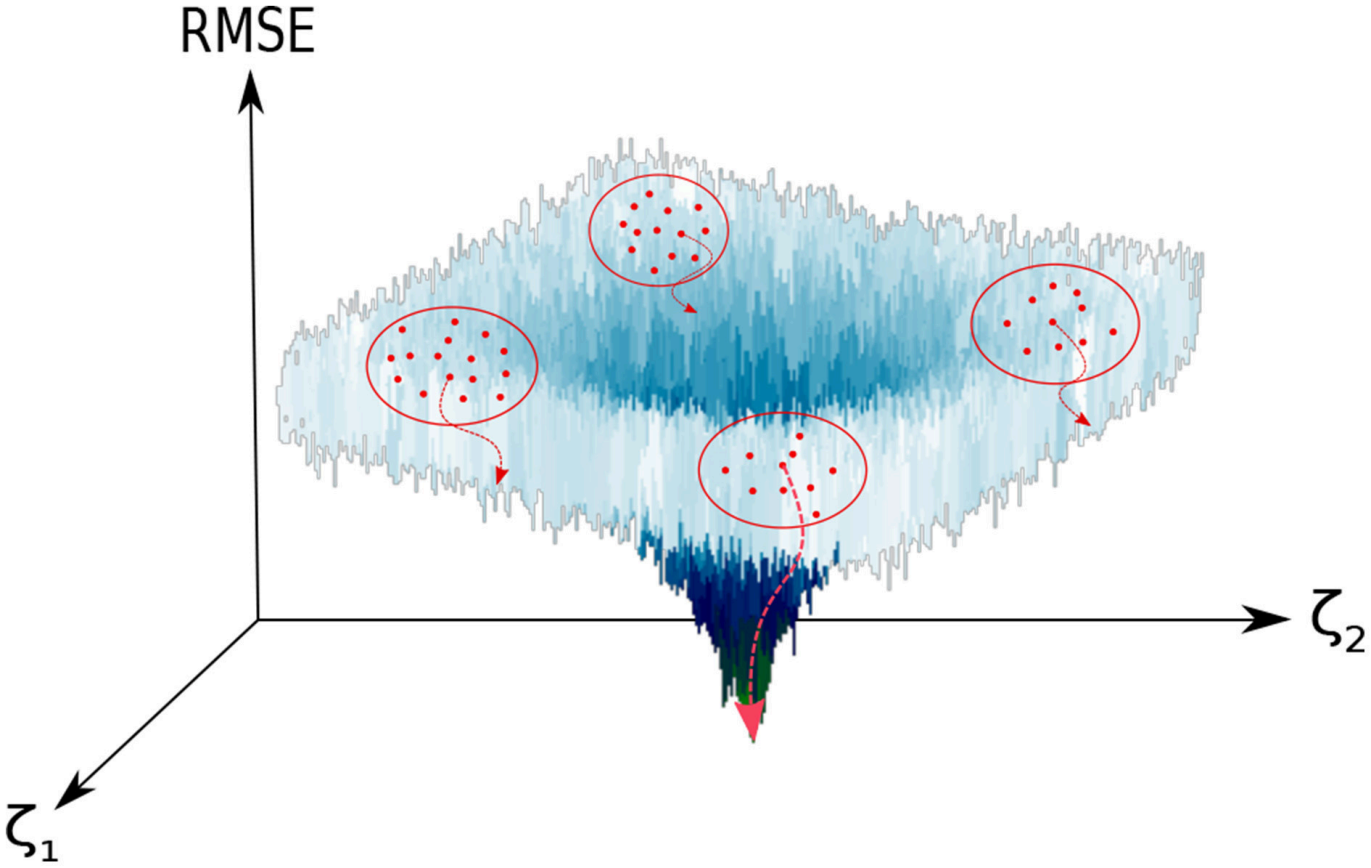
Graphical representation of the optimization process of the set of SRP parameters ({**ζ**}). The vertical axis displays the RMSE between our reference data and our target function (see Equation 3), which in the figure depends just on two parameters (ζ_1_, ζ_2_). Non-overlapping clusters (red dots enclosed in a red circle) of walkers (red dots) are generated. In each cluster, the *optimal* solution is locally minimized (red dotted curved arrows) and compared to the rest of solutions. For a large enough number of clusters, convergence to the global minimum is guaranteed. In this representation, we have used a modified Ackley function (Ackley, 1987).

The problem that concerns us is thus the global optimization of a deterministic non-linear objective function χ(**ζ**): ℝ^*D*^ → ℝ, Equation (1), with a bounded parameter space ($\zeta_{i}\in \left[\zeta_{i}^{min},\zeta_{i}^{max}\right],i=1,\ldots ,D$). In our specific case, we do not make use of the derivatives of this target function since: (i) the analytical expressions are unavailable; (ii) their numerical determination would be expensive and, more importantly, complicated due to the highly-corrugated character of the RMSE landscape (see Figure 1). We shall consider then a *derivative-free optimization* algorithm (Rios and Sahinidis, 2013). As general expression of the objective function (χ) we have considered a *rms-like* function (see Equation 1) composed by two terms: (i) a first one accounting for the error in the energies and (ii) a the second one corresponding to the error in the harmonic frequencies of the stationary points of the PES, with respect to our reference calculations. We have observed that the inclusion of the latter helps to preserve the correct topography of the PES, for instance the first order saddle point character of transition states.

(1) $$\begin{array}{r} \chi_{0}\left(\zeta \right)=\sqrt{\overset{n}{\underset{i=1}{\sum }}\frac{\omega_{E}\left(E_{i}^{ab}\right)\cdot {\left[E_{i}^{ab}-E_{i}^{srp}\left(\zeta \right)\right]}^{2}}{n}+\overset{m}{\underset{j=1}{\sum }}\frac{\omega_{F}\left(\Delta F_{j}\right)\cdot {\left[F_{j}^{ab}-F_{j}^{srp}\left(\zeta \right)\right]}^{2}}{m}} \end{array}$$

where **ζ** is a vector containing the semiempirical parameters and *n, m* represent the number of (relative) energy data points (*E*^*ab*/*srp*^) and harmonic frequencies (*F*^*ab*/*srp*^), respectively, the labels referring to *ab initio* (*ab*) and semiempirical (*srp*) data. The weighting functions $\omega_{E}\left(E_{i}^{ab}\right)$ and ω_*F*_(Δ*F*_*j*_) (with $\Delta F_{j}=F_{j}^{ab}-F_{j}^{srp}$) have been defined as exponential step functions:

(2) $$\begin{array}{r} f\left(x\right)=\{\begin{array}{cc} 1 & x\leq \alpha \\ e^{\beta \left(x-\alpha \right)} & x>\alpha \end{array} \end{array}$$

where α, β are parameters adjusted *a priori* and *x* corresponds to the selected argument ($E_{i}^{ab},\Delta F_{j}$). However, in practice, we have obtained satisfactory results with a much simpler expression:

(3) $$\begin{array}{r} \chi_{1}\left(\zeta \right)=\sqrt{\overset{n+m}{\underset{i=1}{\sum }}\frac{\omega_{G}\left(G_{i}^{ab}\right)\cdot {\left[G_{i}^{ab}-G_{i}^{srp}\left(\zeta \right)\right]}^{2}}{n+m}} \end{array}$$

where *G*_*i*_ = *E*_*i*_||*F*_*i*_ are the components of a vector constructed by concatenating the vectors of energies and harmonic frequencies, respectively. As strategy, we have performed a global optimization step followed by local optimizations in order to refine the results. For the former, we used the Multi-Level Single-Linkage (MLSL) algorithm (Kan and Timmer, 1987) and for the latter we used the Bound Optimization BY Quadratic Approximation (BOBYQA) (Powell, 2009).

### 2.2. Automated Generation of the Set of Reference Points

In the following, we shall describe our automated methodology for the generation of a set of fitting points for the reparametrization of a semiempirical Hamiltonian. In brief, we propose the use of the *whole* set of stationary points of a given PES, the so-called RXN (Martínez-Núñez, 2015a,b; Kopec et al., 2019), as initial set from which neighboring geometries spanning the region of configuration space of interest will be generated. The main advantage of our method is that starting from a *single* initial input geometry, a *global* Potential Energy Surface is generated.

**Figure 2:**
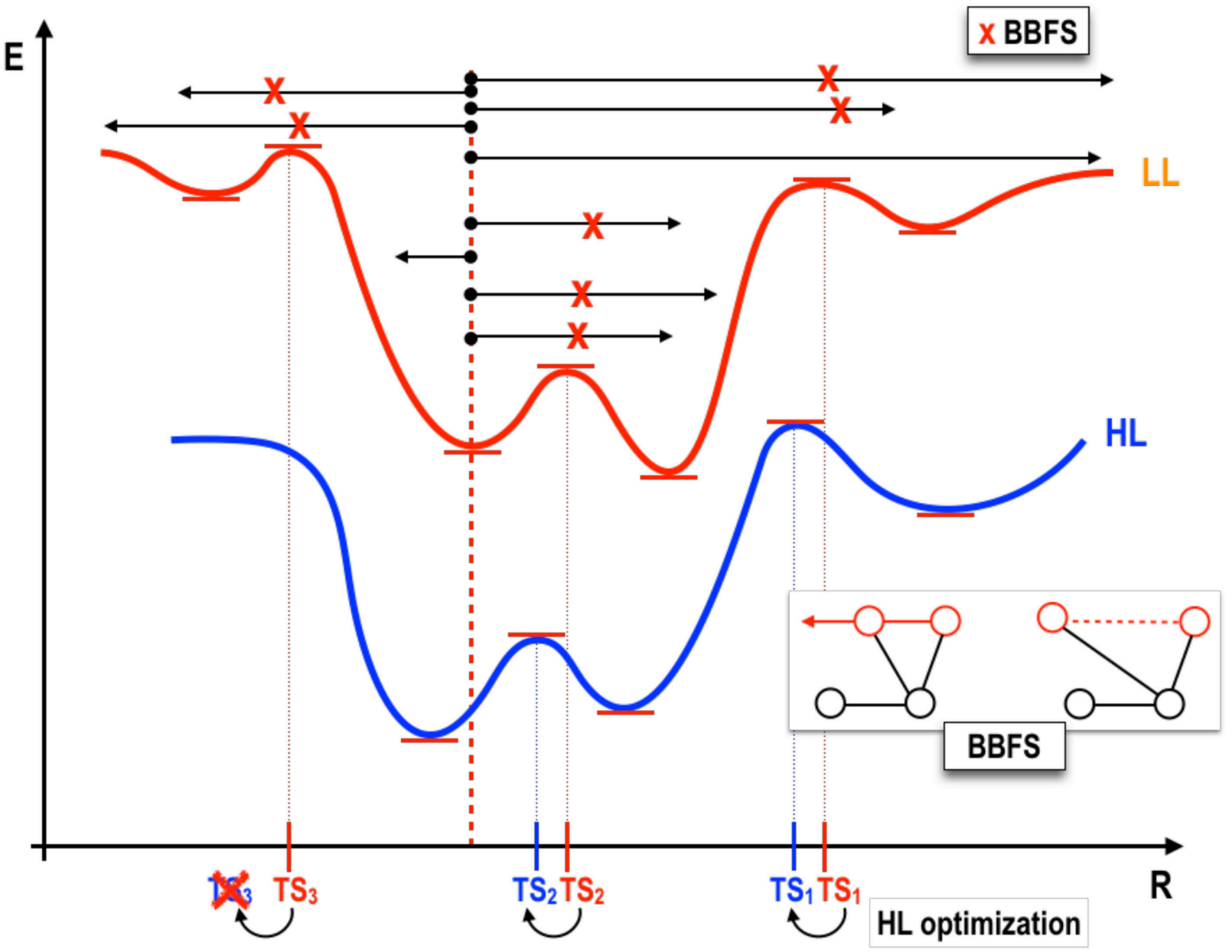
One-dimensional representation of the TSSCDS procedure. A low level (LL) PES (upper energy curve, in red) is sampled starting from a given minimum (geometry indicated by a red dotted line). Classical random trajectories (black arrows) in combination with a graph theory based method (Bond Breaking/Formation Search, BBFS Martínez-Núñez, 2015a,b) lead to the determination of TS candidate structures (marked as x in red bold font), compatible with the total energy of the trajectories, from which LL optimizations are started. Subsequent optimization at the desired high-level (HL) are performed using the LL TS as guess structures.

We propose as first step the determination of the ensemble of stationary points (RXN) on a given PES which will be used as seed for the subsequent generation of the remaining fitting points. Indeed, the stationary points correspond to the molecular configurations which carry the most relevant topographical information of a given PES and, as such, make ideal candidates for fitting purposes. Finding stationary points, however, is a very tedious task which heavily relies on large amounts of chemical intuition. Fortunately, a family of methods for the automated determination of the RXN has been recently proposed, the so-called Transition State Search Using Chemical Dynamics (TSSCDS) (Martínez-Núñez, 2015a,b) as well as its generalization, vdW-TSSCDS (Kopec et al., 2019). The former is optimal for the study of unimolecular processes whereas the latter has been specifically designed to study non-covalently bound systems. The workflow in both cases is analogous (see Figure 2) and the difference lies in the way transition states (TSs) are characterized. Starting from an initial random geometry (or small set thereof), a large number of high-energy classical trajectories is run using a low-level (LL) of electronic structure theory (semiempirical in our case, other methods are also possible) to compute the forces. The geometries along these trajectories are analyzed by a graph-theory based algorithm (Bond Breaking/Formation Search, BBFS Martínez-Núñez, 2015a,b; Kopec et al., 2019) which detects conformations in which bonds are broken and/or formed. It should be highlighted that this step is precisely what determines the difference between TSSCDS and vdW-TSSCDS. In the former, a square connectivity matrix based on covalent distances is defined, whereas in the latter this matrix takes block-diagonal form and includes both covalent and non-covalent (van der Waals) distances, thus allowing for the determination of non-covalent saddle points. The so-determined structures, candidates to TSs, are optimized at the LL and subsequently reoptimized at an appropriate higher level of theory, say, *ab initio* or DFT. Obviously, this process can be continued by further refinements. From this set of final high-level TSs, IRC calculations connecting minima are performed. And, as a result of this, the so-called Reaction Network (RXN) is obtained, that is, all stationary structures together with their connectivities compatible with a given total energy (that of the initial classical trajectories). For further details on the method, the interested reader is referred to the original publications (Martínez-Núñez, 2015a,b; Kopec et al., 2019). As indicated, the RXN will serve us as initial set from which the full set of fitting points will be generated. The total number of stationary points (*N*_*RXN*_) is:

(4) $$\begin{array}{r} N_{RXN}=n_{min}+n_{TS}+n_{asymp}+\ldots , \end{array}$$

where *n*_*X*_, (with X=min, TS, asymp,…) is the number of minima, transition states (TS), asymptotic products, respectively. This initial set will be extended by systematically adding a set of *neighboring* geometries. This can be achieved in different ways. In our case, we have chosen to distort each of the *N*_*RXN*_ points following an n-body type of scheme inspired by a previous work (Pradhan and Brown, 2017). The novelty of our procedure lies in the fact that we observe convergence in the RMSE at each order of the expansion. As it will be clear later, this convergence provides us with an efficient error control and allows to determine a minimal number of fitting points necessary to achieve a given RMSE. The total number of fitting points (*N*_*ref*_) can be calculated as:

(5) $$\begin{array}{rcl} N_{ref} & = & N_{RXN}\cdot \left[\overset{f}{\underset{i\in 1D}{\sum }}N_{i}^{\left(1D\right)}+\overset{f}{\underset{i\in 2D}{\sum }}N_{i}^{\left(2D\right)}+\ldots \right]+rnd\left(fD\right)\\ \; & \; & +\overset{n_{TS}}{\underset{i}{\sum }}N_{i}^{IRC}+\overset{n_{asymp}}{\underset{i}{\sum }}N^{\left(asymp\right)}+\ldots \end{array}$$

where *f* is the number of degrees of freedom of the molecular system, *N* is the number of generated reference geometries of a given type, for instance, *N*^*nD*^ are grid points from a n-dimensional (D) grid and $N_{i}^{IRC}$ are the IRC points stemming from *TS*_*i*_, *rnd*(*fD*) are random geometries in the full-D configuration space, *n* is the number of stationary points of a kind. Considering, for instance, a normal mode or internal coordinate local representation, 1D would refer to displacements along each mode/coordinate (leaving the remaining coordinates fixed at their equilibrium values) and nD refers to grids of points generated through simultaneous displacement along n modes/coordinates, leaving the remaining fixed as before.

Our goal is now to determine the minimum number of fitting points leading to the smallest possible RMSE (defined as the difference between reference PES and SRP-PES), or, in other words, the *optimal* set of SRP parameters ({ζ_*opt*_}). It should be emphasized that we are dealing with moderate-size configuration spaces, in our specific case HONO (6D), the parameter space is 34-dimensional. Hence, in order to systematically search for the global minimum in SRP parameter space ({ζ}), we increase the number of reference points in a controlled way according to the following prescription. Starting with the PM7 parameters ({ζ_*PM*7_}) as initial guess, the RMSE(**ζ**) landscape is explored in a first stage using a small number of ab initio reference data and a big number of iterations (typically of the order of 10^5^) of the non-linear optimization algorithm (MLSL in our case). This allows to locate the most-likely candidate parameter set to global minimum. The latter is used as a guess in subsequent local optimization stages (BOBYQA). At each of these, extended sets of points are generated in the form of *nD* distortions. At each level (1D, 2D, etc.) and for each set, we carry out local optimizations, compare the resulting RMSEs and take as *optimal* the number of points (set) that leads to a satisfactory value of RMSE, in the form of convergence, thus guaranteeing the condition of minimum number of points.

### 2.3. Generation of the SRP-MGPF Potential Energy Surface

As any other grid-based method, MCTDH quantum dynamics relies on a discretization of the configuration space known as *primitive* grid (Kosloff, 1988). In an *f*-dimensional molecular system (typically *f* = 3N-6, with N being the number of atoms), a set of *i*_κ_ = 1, …, *N*_κ_ grids points is defined for the κ-th DOF with κ = 1, …, *f*. In other words, a given grid point *I* ≡ (*i*_1_, …, *i*_*f*_) has an associated molecular configuration (*Q* ≡ (*q*_*i*_, …, *q*_*f*_)). The wavefunction in MCTDH is expressed in a two-layer scheme, a first one in terms of time-dependent single-particle basis functions (SPFs, {**φ**^(κ)^}):

(6) $$\begin{array}{r} \text{Ψ}\left(q_{1},\ldots ,q_{f},t\right)=\underset{j_{1}}{\sum }\ldots \underset{j_{f}}{\sum }A_{j_{1}\cdots j_{f}}\left(t\right)\;\overset{f}{\underset{\kappa =1}{\prod }}\varphi_{j}^{\left(\kappa \right)}\left(q_{\kappa },t\right) \end{array}$$

and a second in which each SPF is, in turn, expressed in a time-independent basis set ($\left\{\chi^{\left(\kappa \right)}\left(q_{\kappa }\right)\right\}$):

(7) $$\begin{array}{r} \varphi_{j_{\kappa }}^{\left(\kappa \right)}\left(q_{\kappa },t\right)=\overset{N_{\kappa }}{\underset{i_{\kappa }=1}{\sum }}c_{j_{\kappa }i_{\kappa }}^{\left(\kappa \right)}\left(t\right)\chi_{i_{\kappa }}^{\left(\kappa \right)}\left(q_{\kappa }\right) \end{array}$$

the latter, typically, Discrete Variable Representation (DVR) functions (Beck et al., 2000; Light and Carrington, 2000). In this frame, each grid point *i*_κ_ (κ-th DOF, *q*^(κ)^) is associated to a localized time-independent basis function (χ^(κ)^(*q*^(κ)^)). Obviously, a minimum number of basis functions, or conversely grid points must exist to achieve the numerical convergence of a given calculation. Such grid representations imply that quantities, particularly the PES, are represented by *f*-dimensional *tensors*, where *f* is the number of DOF. If each DOF is represented by 10 grid points, a tensor of 10^*f*^ grid points would be necessary to represent the PES. It should be clear at this point that that generation of such a high-dimensional PES tensor directly from electronic structure (i.e., quantum chemistry) codes is, nowadays, a prohibitively-long process.

Apart from diminishing the computational time associated to each quantum chemical calculation, solutions to this issue must imply a reduction in the number of grid points necessary to achieve an accurate grid representation of the PES. This can be achieved in two ways. When considering a more or less localized region of the PES (i.e., centered around a given minimum), local approaches such as the Quartic Force Field representation (QFF) can be used. This is the case when computing vibrational eigenenergies and/or eigenstates (Barone, 2005; Ávila and Carrington, 2009; Neff and Rauhut, 2009). On the other hand, when more global representations are needed (e.g., spectroscopy in multi-well problems, reactivity, etc.) one has to resort to more elaborated forms such as tensor-decomposition algorithms (Kolda and Bader, 2009) or Neural Networks (NN) representations (Manzhos et al., 2006). Two examples of this have been recently proposed for a 6D problem (HONO). With respect to the former, Baranov and Oseledets have used a Tensor-Train tensor-decomposition approach (Baranov and Oseledets, 2015) and Pradhan and Brown have illustrated the use of an exponential NN *ansatz* to represent the same PES (Pradhan and Brown, 2017). In both cases, the number of data-points (i.e., high-level *ab initio* calls) needed to perform the fit was of the order of ~10^4^. Upon an increase of the dimensionality of the problem, this last figure is expected to increase, at least, polynomically, hence preventing the use of these techniques for larger systems.

Our method deals with the aforementioned issues by combining an extremely efficient level of electronic structure, a reparametrized semiempirical Hamiltonian, with an efficient and accurate tensor decomposition scheme, Multigrid POTFIT (MGPF) (Peláez and Meyer, 2013). This tensor decomposition algorithm transforms a multidimensional function (e.g., PES) into Tucker product form (Equation 8) in an *quasi* black-box manner. MGPF, implemented in the MCTDH software package (Worth et al., 2016), avoids running over the full (primitive) MCTDH grid and, instead, uses a series of coarser (nested) grids using a number of PES data-points comparable to the aforementioned methods. However, the big difference is that in our case we shall perform SRP calls, in other words, our *ab initio* method will have the computational cost of a semiempirical one. In fact, as shown by our results (see Table 1 in section 3.1), we need no more than hundreds of high-level *electronic structure* calls in comparison to the tenths of thousands points required by previous methods. This, obviously, leads to a (small) error inherent to the SRP approximation, but in contrast permits the extension of our approach toward higher-dimensional systems with a little more effort. In the following lines, we shall describe the actual MGPF approach that we have used.

**Table 1:** Number and description of the fitting points used in each SRP-optimization stage and the algorithm used in the process.

**No. points**	**Class of points**	**Type of optimization**
53	*core*	Global/Local
367	1D + *core*	Local
546	1D + 2D + *core*	Local
648	1D + 2D + rnd(6D) + *core*	Local
954	1D + 2D + rnd(6D) + LIIC-IRC + *core*	Local
1084	1D + 2D + rnd(6D) + LIIC-IRC + rnd(LIIC) + *core*	Local

*Structures have been generated within a set of fixed boundaries defined in Table 3. The initial set of geometries (labeled core) consists on 53 points, namely: MIN1, MIN2, TS1, 1D- and 2D-distorted structures using the latter as reference geometries [26 1D, 14 2D], and 10 6D-randomly distorted (rnd(6D)) geometries. The number of points at each new set is cumulative. It includes nD-distorted geometries (n = 1, 2, 6), LIIC structures and distortions thereof [noted as rnd(LIIC)]. The algorithm for global optimization is MLSL and for local is BOBYQA (see section 2.1). The number of iterations in the global step has been set up to 100,000 and in the local one to 2,000*.

In MGPF, we use a sum-of-products or Tucker expansion for the PES:

(8) $$\begin{array}{r} V=\overset{\left[m_{1},\ldots ,m_{f}\right]}{\underset{j_{1},\ldots ,j_{f}}{\sum }}C_{j_{1},\ldots ,j_{f}}\overset{f}{\underset{\kappa =1}{\prod }}\;v_{j}^{\left(\kappa \right)} \end{array}$$

which, in tensor notation, can be written as: Kolda and Bader (2009)

(9) $$\begin{array}{r} V=C\times_{1}v^{{\left(1\right)}^{T}}\times_{2}v^{{\left(2\right)}^{T}}\cdots \times_{f}v^{{\left(f\right)}^{T}} \end{array}$$

There C is the so-called *core* tensor and ***v***^(κ)^ are the expansion basis sets for the κ-th DOF. The reader is referred to the original article for a full description of the method and its capabilities (Peláez and Meyer, 2013). More specifically, our current application uses a bottom-up approach to MGPF (Peláez and Meyer, in preparation). The MGPF basis sets ({***ṽ***^(κ)^}) can be expressed as:

(10) $$\begin{array}{r} {\tilde{v}}^{\left(\kappa \right)}=\rho^{{\left(\kappa \right)}^{\prime }}\rho^{{\left(\kappa \right)}^{-1}}v^{\left(\kappa \right)}\;. \end{array}$$

There we have introduced potential density matrices of the form: Peláez and Meyer (2013)

(11) $$\begin{array}{r} \rho_{kk^{\prime }}^{\left(\kappa \right)}:=\underset{I^{\kappa }}{\sum }V_{I_{k}^{\kappa }}\;V_{I_{k^{\prime }}^{\kappa }}\;\kappa =1,\ldots ,f\;. \end{array}$$

where the first index (*k*) runs along the primitive grid in ***ρ***^(*κ*)′^ and along the coarse one in ***ρ***^(*κ*)^. The transpose of these basis sets reads then:

(12) $$\begin{array}{r} {\tilde{v}}^{{\left(\kappa \right)}^{T}}=v^{{\left(\kappa \right)}^{T}}{\left(\rho^{{\left(\kappa \right)}^{\prime }}\rho^{\left(\kappa \right)-1}\right)}^{T} \end{array}$$

Substituting in the MGPF expansion *V*^MGPF^ of the form Equation (9), we unitarily transform both the MGPF basis set (**ṽ**) and the MGPF *core* tensor (C) using the complete basis ***v***: Peláez and Meyer (in preparation)

(13) $$\begin{array}{r} {\tilde{V}}^{\text{MGPF}}=C\times_{1}\left(v^{{\left(1\right)}^{T}}v^{\left(1\right)}\right){\tilde{v}}^{{\left(1\right)}^{T}}\times_{2}\left(v^{{\left(2\right)}^{T}}v^{\left(2\right)}\right){\tilde{v}}^{\left(2\right)}\;\cdots \times_{f}\left(v^{{\left(f\right)}^{T}}v^{\left(f\right)}\right){\tilde{v}}^{\left(f\right)} \end{array}$$

It should be noted that this transformation does not change the representation. Then one obtains:

(14) $$\begin{array}{r} {\tilde{V}}^{\text{MGPF}}=V\times_{1}{\tilde{\gamma }}^{{\left(1\right)}^{T}}\times_{2}{\tilde{\gamma }}^{{\left(2\right)}^{T}}\cdots \times_{f}{\tilde{\gamma }}^{{\left(f\right)}^{T}} \end{array}$$

where V is the tensor of the energies on the coarse grid and ${\tilde{\gamma }}^{\left(\kappa \right)}$=***ρ*^(*κ*)′^*ρ*^(*κ*)−1^** is the new MGPF basis set. Both quantities, *core* tensor (V) and potential density matrices are directly computed by interfacing the MGPF routine of MCTDH to the openMOPAC software package.

### 2.4. Calculation of Vibrational Properties: Eigenenergies and Eigenstates

To provide a stringent test to the quality of our series of *chemically accurate* SRP-PES, in addition to RMSEs we have also computed ground and vibrationally excited eigenstates and compared them to those of the reference PES (Richter et al., 2004). These vibrational calculations have been computed using the Heidelberg version of the MCTDH software package (Worth et al., 2016) using our SRP-MGPF PES, as described above. It should be highlighted that the problem we are considering (HONO) features a double well and, consequently, single-reference approaches (e.g., QFF) are not well-suited to its study.

The calculation of the vibrational eigenstates and eigenenergies has been performed by propagating a guess WF in negative imaginary time using the so-called Improved Relaxation method (Meyer and Worth, 2003; Meyer et al., 2006). The MCTDH equations of motion (EOM) are here obtained through a time-independent variational principle. As a result, the propagated configuration interaction coefficients (*A*, see Equation 6) are obtained through diagonalization of the Hamiltonian in the basis of the configurations:

(15) $$\begin{array}{r} \underset{L}{\sum }\langle \Phi_{J}|H|\Phi_{L}\rangle A_{L}=EA_{J}\;, \end{array}$$

and the single-particle basis functions (SPFs) are evolved in imaginary time using the *standard* MCTDH EOM (Beck et al., 2000). This iterative procedure is repeated until convergence in the energy. Moreover, a block version of this algorithm, the so-called Block Improved Relaxation, can be used to converge several eigenstates simultaneously, thus leading to the determination of a set of vibrationally excited states.

## 3. Results and Discussion

In this section, we present the application of the SRP-MGPF methodology to the actual computation of the HONO (6D) PES for the *cis-trans* isomerization region, which has become a benchmark for this type of studies (Baranov and Oseledets, 2015; Pradhan and Brown, 2017). In the following subsections, we shall discuss the details on the generation of the fitting reference set of points, the reparametrization of the semiempirical Hamiltonian (SRP), and the technical details concerning the direct MGPF tensor decomposition of the SRP-PES into Tucker form. It should be stressed that the novelty and robustness of our approach resides in the fact that requires a minimum intervention of the user, thus qualifying as a *quasi*-black box approach. For the time being, we have interfaced the software openMOPAC to the MCTDH software package through the use of the MGPF tensor decomposition algorithm (Peláez and Meyer, 2013), hence allowing quantum dynamical simulations on a SRP-MGPF PES.

### 3.1. Computation of the SRP-MGPF PES for the *cis-trans* Isomerization Region in the HONO System (6D)

**Figure 3:**
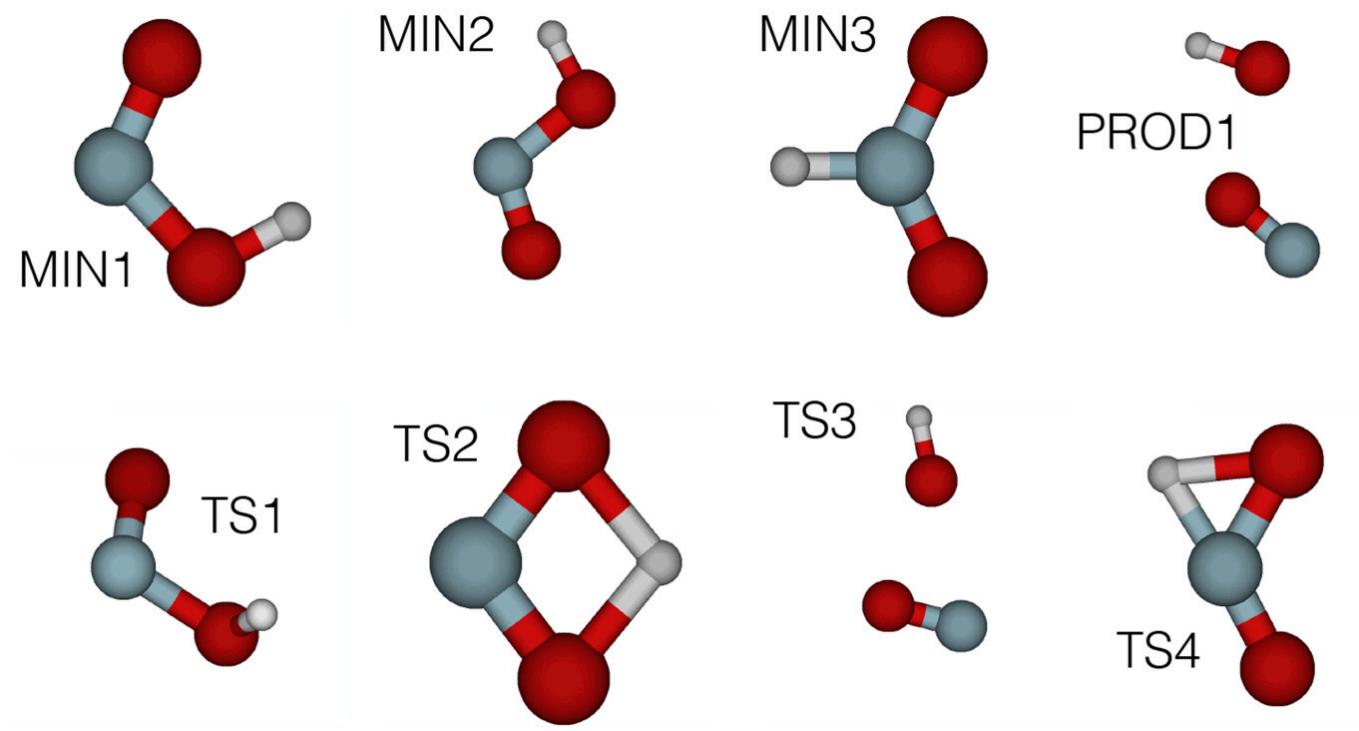
MP2/cc-pVDZ (intermediate HL) structures of HONO automatically obtained using the TSSCDS algorithm on a PM7 (LL) PES. Target geometries in the *cis-trans* isomerization region (MIN1, MIN2, TS1) were subsequently reoptimized at the CCSD(T)/cc-pVQZ (final HL) level of theory.

The first stage in our automated fitting procedure has been the determination of the stationary points of HONO, accomplished through the use of the TSSCDS package (Barnes et al., 2019), as described in section 2.2. Starting from a single random input geometry, LL guess structures have been obtained (see Martínez-Núñez, 2015a,b for a detailed discussion). Figure 3 presents the corresponding MP2/cc-pvDZ structures. The relevant geometries for our study *cis* (MIN1), *trans* (MIN2) as well as the TS connecting them (TS1) have been reoptimized at the CCSD(T)/cc-pVQZ level of theory. Their geometrical parameters and harmonic frequencies are presented in Tables S10–S13. The reason behind the choice of this level of theory is that we have taken as model chemistry the CCSD(T)/cc-pVQZ quality analytical PES of Richter et al. (2004)

The generation of the remaining reference geometries and corresponding energies has been done according to our heuristic approach described in section 2.2. A set of geometries in the form of *n*D-product grids (*n*=1, 2) and 6D-random structures have been generated using the three lowest energy stationary points of HONO as pivotal geometries, namely: *cis, trans*-conformers and the corresponding TS (see Figure 3: MIN1, MIN2, and TS1, respectively). Moreover, the reaction path among them has been taken into account through a piecewise Linear Interpolation in Internal Coordinates (LIIC) (Soto et al., 2006) between the *cis*-TS and TS-*trans* pairs of stationary points (see Figure S1) as well as a cloud of distorted structures around them. To ensure that the latter remain close to the *reaction path* (LIIC), each *i*-th geometry along the LIIC has been generated by distorting along a set of directions resulting from the linear combination of the normal modes of the end structures according to:

$$\begin{array}{l} \Delta {\vec{Q}}_{i}=\left(1-X_{i}\right)\cdot {\vec{Q}}_{init}+X_{i}\cdot {\vec{Q}}_{fin}\;\vec{Q}\in {\mathbb{R}}^{3N-7} \end{array}$$

where ${\vec{Q}}_{fin}=$TS1, ${\vec{Q}}_{init}=$MIN1/MIN2. *X*_*i*_ is a number that depends on the *distance* to the end structure. The closer to ${\vec{Q}}_{fin}$ the more $\Delta \vec{Q}$ resembles the normal modes of the end structure (TS1). Each of our LIIC consists of 50 points and the aforementioned *distance* is simply taken as the ordinal *i* within the LIIC. It should be noted that the torsion mode has not been included (3*N*−7 modes in total), since it approximately corresponds to the reaction coordinate. Finally, for a given displacement ($\Delta \vec{Q}$), the geometries around the *i*-th geometry along the LIIC have been computed as:

$$\begin{array}{l} {\vec{R}}_{i}={\vec{R}}_{i}^{\left(0\right)}+\overset{3N-7}{\underset{j=1}{\sum }}f_{j}\cdot \Delta {\vec{Q}}_{i,j} \end{array}$$

where ${\vec{R}}_{i}^{\left(0\right)}$ is the original geometry of the *i*-th point of the LIIC, *f*_*j*_ is a small random factor, and $\Delta {\vec{Q}}_{i,j}$ is the *j*-th component of $\Delta {\vec{Q}}_{i}$.

This systematic manner of generating reference points serves us to control the convergence of the RMSE error at each expansion order, in other words, how insensitive the RMSE is to an increase in the density of points in specific directions (or combinations thereof). This, in turn, provides us with a good estimate of the *lowest possible* number of reference geometries at each stage. In Table 1, we present the different convergence stages in terms of number of fitting points used together with the associated optimization algorithm. As it can be observed, at each specific stage, we either increase the density of points in the indicated directions (*modes/coordinates*) or add a new class of points in the form of a LIIC, for instance.

**Figure 4:**
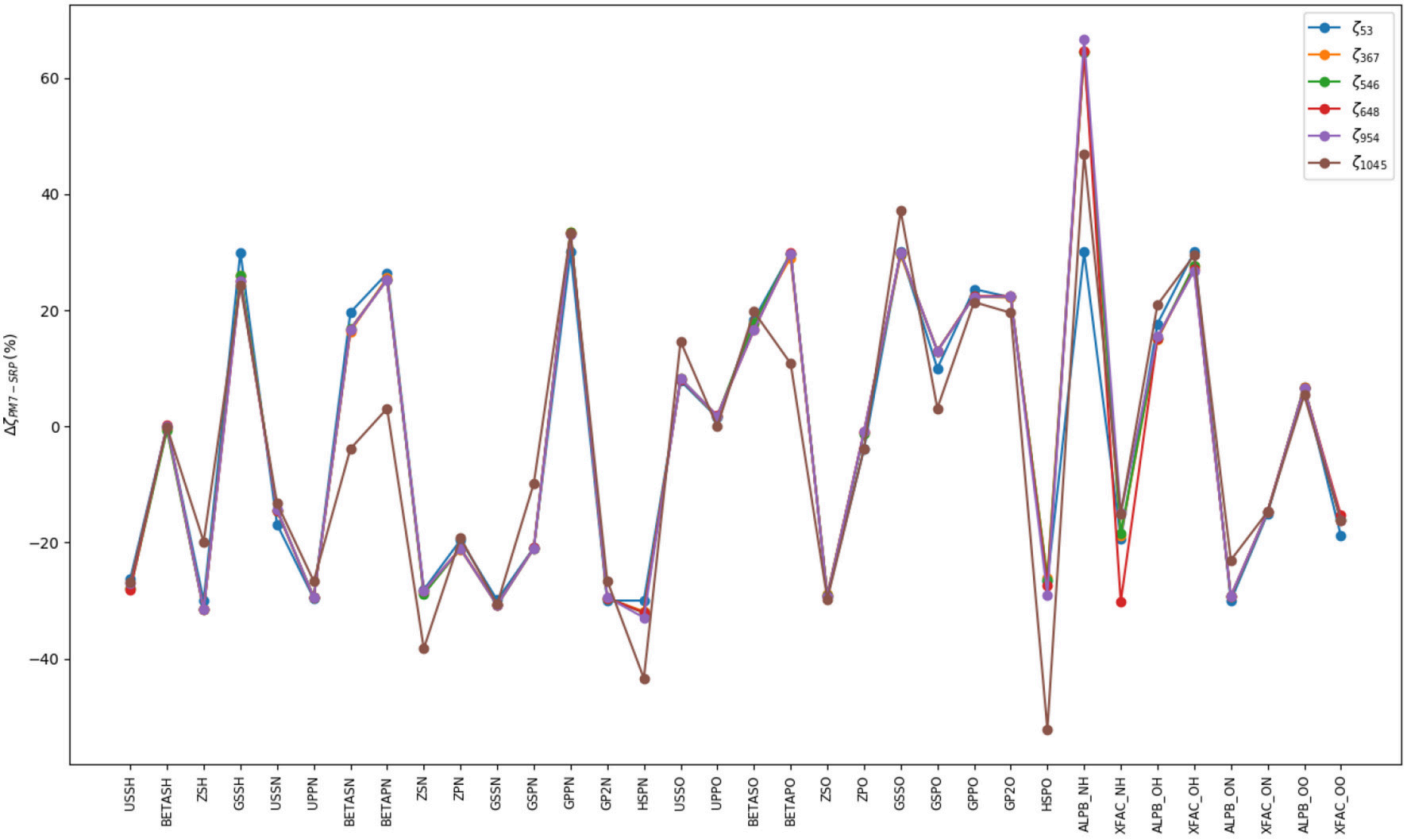
Percentage of variation of the SRP parameters with respect to the original PM7 ones. Each fitting stage is represented by its *optimal* parameters, ζ_*N*_, where N is the number of points used in the process (see Table 1). On abscissas we present the label of semiempirical parameters for the different type of atoms in HONO. Standard semiempirical parameter labeling has been used (Stewart, 2013). Parameters from USSH until HSPO correspond to a single type of atom whereas parameters labeled ALPB_*XY*_ and XFAC_*XY*_ correspond to two-atom ones (atom X and atom Y).

**Figure 5:**
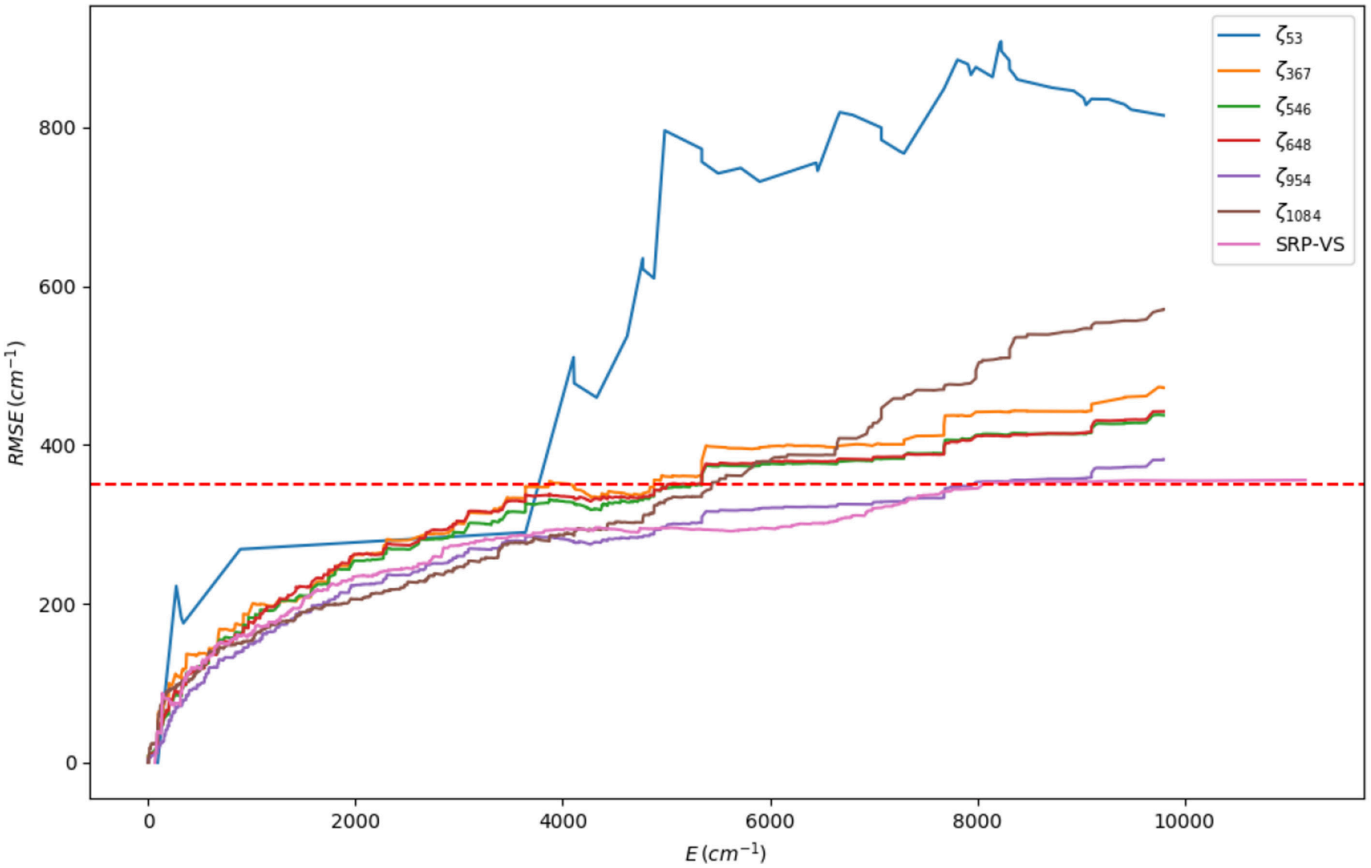
Cumulative RMSE for each SRP-fit labeled by its set of parameters, ζ_*N*_, where N is the number of points used in the fit (see Table 1). The last set (ζ_*VS*_) corresponds to the validation set. The red dotted horizontal line represents the value of the chemical accuracy (1 kcal/mol≈ 350 cm^−1^).

The first stage consists on a global optimization (MLSL) followed by a local one (BOBYQA) using a small number of judiciously chosen points: the RXN and a cloud of random geometries around them, adding up to a total of 53 points. This has enabled a very large number of iterations (10^5^). The underlying hypothesis behind this calculation is that a reasonable and cheap estimate of the *global minimum* (set of SRP parameters yielding the minimum RMSE) can be obtained. Our best set of parameters at this stage (**ζ**_53_, where 53 is the number of fitting points) yielded an initial RMSE of 806.8 cm^−1^ (Table S1). In the subsequent stages, we have performed local optimizations (BOBYQA) with 2,000 iterations. Before proceeding any further, we would like to justify the use of a global algorithm exclusively at the first stage, in other words, ζ_53_ must indeed correspond to a set near the *global* minimum or a local deep minimum. First, from a computational perspective, it should be noted that a small number of fitting points is ideally suited for this task. Second, we have performed calculations justifying this fact. Table S2 (column 2) presents the BOBYQA variation of the RMSE for an increasing number of 1D-sets of fitting points. It can be observed that upon increase of this number, from 192 until 2088 fitting points, the RMSE monotonically decreases from 482.13 cm^−1^ till 365.13 cm^−1^. According to our reasonings above, one should take the SRP parameters of the last set of points (**ζ**_1542_ or **ζ**_2088_) corresponding to the best RMSE of the 1D-series. For the sake of efficiency, we considered the **ζ**_1542_. With this set of SRP parameters, we recomputed the whole series of RMSEs for the different sets of 1D-points and we observed a very close agreement with the BOBYQA values, except for the 192 set. This shows that indeed all sets of parameters of this series (from **ζ**_367_ on) lie within the *same* RMSE landscape region (see Figure 4) and, in turn, validates our initial approach with a small number of *representative* points. One can then safely conclude that just 367 fitting points are necessary to improve the SRP-fitting at the 1D-level. Hence, subsequent 2D optimizations will start with the (**ζ**_367_) set. A detailed description of all stages and RMSE values is presented in Tables S1–S9. A somewhat more complete information can be obtained through the cumulative error computed by addition of the RMSEs resulting form the configurations up to a certain energy value (see Figure 5). It can observed that for all sets of parameters, with the exception of **ζ**_53_, the RMSEs remain below the limit of chemical accuracy (1 kcal/mol≈ 350 cm^−1^) within the targeted PES region (*cis-trans* isomerization). Moreover, in the last stage we have removed all structures with energies above 5000 cm^−1^ (above the classical barrier) and included an extra set of random points around the stationary points. This new set of points has been used to BOBYQA reoptimize the SRP. We observe a clear improvement of the RMSE in such a way that, up to 8000 cm^−1^, the RMSE is inferior to the chemical accuracy level. The correctness of these results has been supported by a calculation using a validation set consisting of 1200 6D random points with energies below 12000 cm^−1^ for which the same pattern is obtained. We have also compared the geometries and harmonic frequencies of all stationary points at the reference *ab initio* level of theory and at the SRP level for each stage. Geometries are displayed in Tables S10–S12 and harmonic frequencies are shown in Table 2. As it can be observed, SRP does indeed improve, in terms of both geometrical parameters and harmonic frequencies, with respect to the original PM7 and, furthermore, we obtain a very good agreement with the reference *ab initio* data. This is particularly true for the last stage (**ζ**_1084_).

**Table 2:** Harmonic frequencies of the normal modes of each stationary point at the CCSD(T)/cc-pVQZ *ab initio* level of theory and corresponding values for the PM7 method and the SRPs in the different stages of the optimization.

	**Harmonic frequencies (cm^−1^)**							
	***Ab initio***	ζ_PM7_	ζ_53_	ζ_367_	ζ_546_	ζ_648_	ζ_954_	ζ_1084_
TS	-599.2	-553.6	-581.0	-565.1	-568.7	-570.3	-573.3	-606.8
	559.1	621.7	512.2	467.1	465.5	463.6	463.8	597.2
	791.2	1021.3	654.7	649.5	652.7	653.6	654.9	738.4
	1122.3	1175.3	1174.3	1092.3	1099.8	1095.8	1106.8	1195.4
	1728.0	1839.5	1763.8	1705.8	1709.4	1710.5	1711.0	1737.9
	3785.3	2801.7	3747.9	3568.9	3585.3	3585.0	3586.4	3736.2
*cis*	648.7	589.0	615.4	613.0	616.1	618.2	619.3	622.5
	687.9	629.2	724.9	712.0	718.4	716.0	718.4	698.9
	901.9	1084.8	745.3	715.4	721.0	721.7	728.4	854.2
	1350.9	1346.0	1316.4	1252.5	1255.9	1253.7	1262.3	1369.5
	1675.5	1823.5	1725.5	1693.2	1696.6	1698.3	1701.6	1719.1
	3632.1	2802.9	3668.9	3504.6	3519.8	3520.0	3521.4	3667.3
*trans*	574.8	455.9	517.1	515.2	517.1	518.1	521.6	540.5
	633.1	609.8	533.3	515.2	519.1	523.7	528.3	602.5
	839.6	1096.0	730.5	736.6	741.7	744.7	748.9	835.1
	1319.3	1308.8	1232.9	1130.0	1136.9	1131.6	1148.4	1264.6
	1732.6	1826.5	1715.9	1666.7	1670.2	1671.9	1674.8	1704.7
	3790.8	2828.3	3815.8	3662.7	3678.9	3680.9	3682.9	3796.1

**Figure 6:**
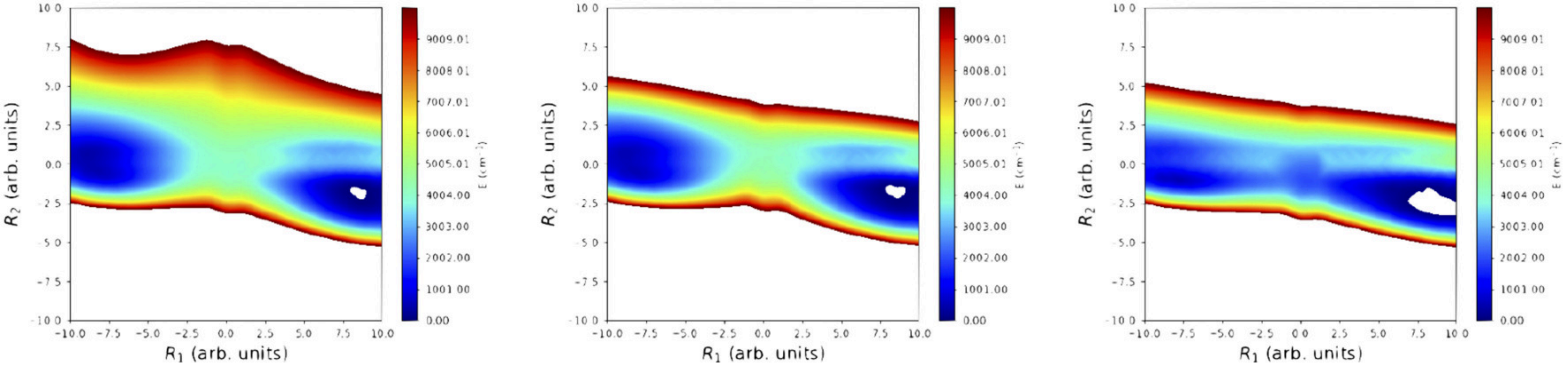
Comparison of the 2D projections of the cis-trans isomerization region for: (i) reference PES (Richter et al., 2004) (left panel); (ii) SRP-PES (ζ_1084_) (middle panel); and (iii) PM7-PES (right panel). These projections have been obtained by orthonormalization of two linear interpolation (LIIC) vectors as described in Soto et al. (2006).

To finalize this section, we present in Figure 6 a comparison of 2D projections of the *cis-trans* isomerization regions for: (i) the reference surface, (ii) the SRP-PES(**ζ**_1084_); and (iii) the PM7 semiempirical Hamiltonian. These contour plots have been obtained through orthogonalization of the two LIIC vectors used in Figure 6. The positive effect of the reparametrization can be clearly observed: while PM7 provides a *blurred* description of the TS region, the SRP-PES reproduces it correctly.

#### 3.1.1. Classical Molecular Dynamics on the SRP-PES

**Figure 7:**
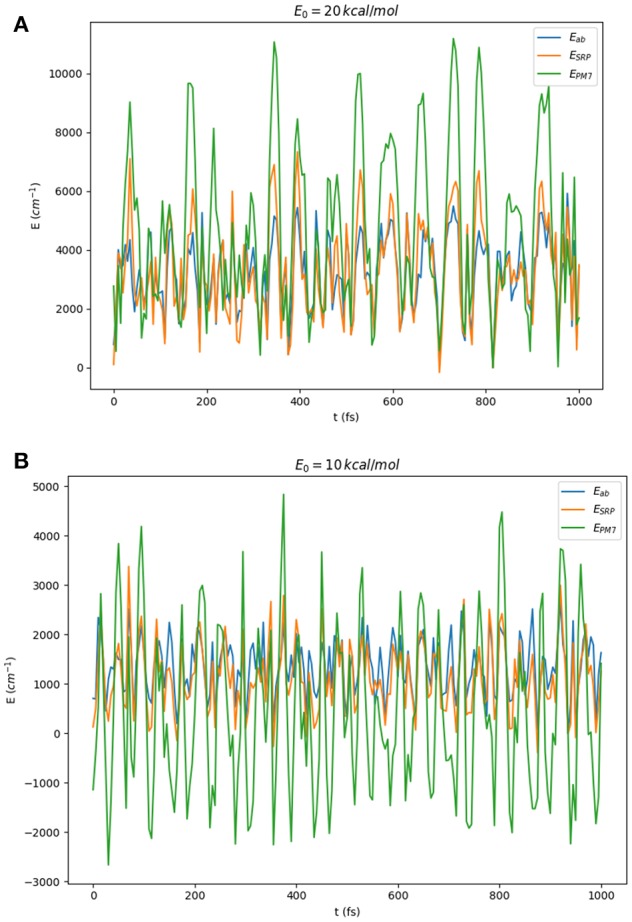
Comparison of ab initio (blue line), PM7 (green line), and SRP-PES (ζ_1084_) (orange line) energies for the geometries generated in classical on-the-fly trajectories of HONO(6D) with total energies (randomly distributed among all modes) of 10 and 20 kcal/mol starting at: **(A)** the *trans*-conformer and **(B)** the *cis*-conformer.

As a first test of the quality of the SRP-PES, we have carried out classical molecular dynamics simulations for the HONO system in full dimensionality using the VENUS96 software package (Hu et al., 1991). Classical trajectories have been run using the reference PES (Richter et al., 2004). The energies of the so-obtained geometries have been subsequently computed at the SRP-PES level and compared to the original calculation. Starting from the equilibrium geometries of the *cis* and *trans* isomers, we have propagated for 1*ps* each trajectory with a time-step of 5*fs*. The vibrational energy of each starting geometry was classically distributed in a random way between all normal modes using the option *normal mode sampling* of the VENUS software. We have computed 10 trajectories per isomer, each isomer having 4 different vibrational energies (5, 10, 15, and 20 kcal/mol) thus making a total of 80 trajectories and 16,080 geometries. In Figure 7, we present a comparison of the variation of the potential energies along two of these trajectories. As it can be observed, the PM7 largely deviates from the reference calculation both in their relative values and the phase, whereas SRP-PES follows closely the *ab initio* values. In particular, it is remarkable the fact that for low energies PM7 presents a large amount of structures with energies below the value of the global minimum, the *trans* conformer. To finalize this subsection, we would like to provide some performance features of the SRP-PES which directly show the efficiency of the underlying openMOPAC software. In the case of the HONO, from an average of the order of ~10^4^ points, we have obtained a mean CPU-time of 10^−2^ s per single-point energy. Moreover, Hessians are computed in less than a second. This properties make SRP approaches suitable for any on-the-fly type of calculation. In particular, we are currently exploring their use with non-grid based quantum dynamical methods such as the Direct-Dynamics Variational Multiconfigurational Gaussian (DD-vMCG) method (Richings et al., 2015).

### 3.2. Full Quantum Analysis of the Vibrational Properties of the SRP-PES for the *cis-trans* HONO System (6D)

To further assess the quality of our SRP-PES we have computed vibrational properties by means of MCTDH quantum dynamical calculations and the results have been compared to the ones from the reference PES (Richter et al., 2004). More specifically, ground and excited vibrational states as well as vibrational spectra, in the form of Fourier transforms of autocorrelation functions. At this point, it should be recalled that our main goal is not to achieve spectroscopical accuracy but to provide PESs, in a fully automated fashion, accurate enough to disentangle chemical processes.

#### 3.2.1. MGPF Tensor Decomposition of the HONO 6D PES

To *interface* the SRP-PES with the MCTDH quantum dynamics software package, we have used the Multigrid POTFIT tensor decomposition algorithm (Peláez and Meyer, 2013). More specifically, all PES *calls* within the MGPF workflow have been addressed directly to the openMOPAC software package using an external set of *optimal* SRP parameters. In other words, at each grid point, i.e., configuration, a SCF process is performed. Of course, this is only possible due to the high efficiency of the underlying PM7 frame. This fact, precisely, has allowed us to circumvent the issues encountered in previous studies in which the *ab initio* energies were generated directly from a quantum chemical calculation thus severely limiting the level of theory which could be applied.

We have carried out *bottom-up* MGPF calculations Peláez and Meyer (2013) to the different SRP-PESs at different parameter optimization stages. In Table S14, we present a comparison in terms of CPU time and memory needs for a reference exact Tucker decomposition (using POTFIT, PF) (Jäckle and Meyer, 1996) and the different MGPF tensor decomposition levels that we have used in this work. The full primitive grid, needed in PF, consists of 2.804· 10^7^ points. In contrast, the coarse grids in MGPFs include every third, fourth, or fifth fine grid point for each DOF. These coarse grids have been labeled *ev*3, *ev*4, and *ev*5 and consist of 172,800, 51,200, and 18,432 coarse grid points, respectively. The MGPF partial grids increase these figures by a factor <10. This is due to the fact that the contracted mode lies fully in the fine grid (see section IIIB in Peláez and Meyer, 2013). Hence, as expected, MGPF is orders of magnitude less demanding that an exact decomposition. The global RMSE values show that MGPF PES are accurate, cheap and, more importantly, add a very small (global, full grid) error to the PES. Finally, it should also be highlighted that none of our SRP-PES present energies below the global minimum (*trans* conformer), whereas the PM7 does. In other words, PM7 presents artificial PES structure when compared to the reference one. We have observed that even the simplest SRP optimization corrects this wrong behavior.

#### 3.2.2. MCTDH Quantum Molecular Dynamics on the SRP-MGPF

**Figure 8:**
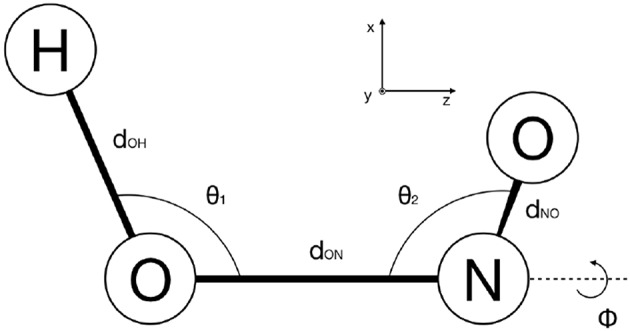
Definition of the internal coordinates of HONO used in this work.

As discussed in section 2.3, MCTDH requires the discretisation of the configuration space. The HONO (6D) molecule has been represented in internal coordinates (see Figure 8) as in previous works (Peláez and Meyer, 2013; Pradhan and Brown, 2017), and a Discrete Variable Representation (DVR) grid has been defined accordingly (see Table 3). We have performed ground and excited eigenstate vibrational calculations for the reference PES, the PM7-MGPF PES as well as for selected SRP-MGPF PES using the Improved Relaxation algorithm and its Block version, as implemented in the Heidelberg version of MCTDH (Meyer et al., 2006). We have combined the physical modes into logical particles as follows: [ϕ=15], [*d*_*OH*_=10] [*u*_2_, *d*_*ON*_=25], [*u*_1_, *d*_*NO*_=25], where the number represents the number of single-particle functions (SPFs) and *u*_*i*_ = cosθ_*i*_ (see Figure 8). In all cases, the initial wave packet has been propagated in *negative imaginary time* (see section 2.4) during 500 fs.

**Table 3:** Definition of the MCTDH primitive grid: HO denotes a harmonic oscillator (Hermite) and cos a cosine Discrete Variable Representation (DVR) basis functions.

**DOF**	**DVR**	**N**	**Range**
*d*_*OH*_	HO	18	[1.30, 2.45]
*d*_*NO*_	HO	13	[1.90, 2.60]
*u*_2_	HO	13	[-0.65, -0.10]
*d*_*ON*_	HO	16	[2.10, 3.25]
*u*_1_	HO	18	[-0.65, 0.25]
*ϕ*	cos	32	[0, 2π/2]

*N is the number of primitive (fine) grid points. The range represents the first and last grid points in atomic units for the distances and ϕ is the torsion angle in radians. Cosines of the valence angles have been used: u_i_ = cos θ_i_. See Figure 8 for the definition. Physical degrees of freedom have been combined into logical modes or particles according to the following scheme: [ϕ], [d_OH_] [u_2_, d_ON_], [u_1_, d_NO_]. The first particle (ϕ) has been contracted in MGPF (see section IIIB in Peláez and Meyer, 2013)*.

With respect to ground state energies, the reference PES yields a value of 4367.7 cm^−1^ for the Zero Point Energy (ZPE) and the PM7-MGPF PES a value of 3221.3 cm^−1^, well off the analytical one. We attribute this discrepancy to the artificial structure of the PES revealed by the presence of *negative* energies (geometries with energies below the global minimum, *trans* conformer) as discussed in section 3.2.1) and clearly illustrated in Figure 7. On the other hand, concerning the SRP-MGPF PESs, a nice convergence can be observed upon increase of the number of fitting points, toward a final value of 4332.8 cm^−1^ which compares well with the analytical one. It is also remarkable that a simple fit using only 53 fitting points already leads to a qualitative improvement with respect to PM7. Moreover, our results show that the ZPE values are somewhat insensitive to the size of the coarse grid (cf. last three rows of Table 4). Consequently, we shall use hereafter the *ev*5 SRP-MGPF scheme.

**Table 4:** Ground state energies of HONO using PESs of different quality.

**Set**	**MGPF**	**ZPE (cm^**−1**^)**
**ζ**_*PM*7_	*ev*4	3221.3
**ζ**_53_	*ev*4	4070.7
**ζ**_648_	*ev*4	4095.0
**ζ**_1084_	*ev*4	4332.8
**ζ**_1084_	*ev*5	4330.8
**ζ**_1084_	*ev*3	4332.9

*The first column indicates the set of SRP parameters used, labeled by its set of parameters, ζ_N_, where N is the number of points used in the fit (see Table 1). The second column presents the size of the MGPF coarse grid: evn indicates a coarse grid in which every (ev) n-th fine grid point has been considered (see section 3.2.1). The final column presents the Zero Point Energies (ZPE) for each of the previous PES*.

We have also computed the 20 lowest-lying vibrational eigenstates of HONO (Table 5). It should be noted that this energy interval spans all HONO fundamentals except the OH stretching mode. For this, we have considered four different PES, namely: (i) PM7-MGPF, SRP-MGPF with **ζ**_53_ and **ζ**_1084_, as well as the reference (exact) PES. The first remark to be done is that the original PM7-MGPF PES fails to predict the initial vibrational state corresponding to the *ground state* of the *cis* conformer (Richter et al., 2004). In contrast, even at the minimum level of reparametrization (**ζ**_53_), this eigenstate is obtained. Furthermore, this incorrect behavior worsens upon increase of the energy. In fact, eigenenergies are off by several hundreds of cm^−1^ in almost the its whole range. This can be readily understood by simple observation of the 2D contour plots of the *cis-trans* region of the PES (see Figure 6). In contrast, both SRP-MGPFs nicely follow the reference values and, what is more important, the discrepancies (of the order of tens of cm^−1^) do not increase but remain, in average, constant.

**Table 5:** Comparison of the 20 lowest vibrational eigenvalues of HONO for different PESs denoted by its set of parameters, **ζ**_*N*_, where N is the number of points used in the fit (see Table 1).

	**Vibrational eigenenergies (cm**^****−1****^**)**			
	ζ_PM7_	ζ_53_	ζ_1084_	**Analytical**
	0.0	0.0	0.0	0.0
	593.6	163.0	88.5	94.1
	794.3	604.7	597.1	600.8
	1070.6	693.2	703.9	710.7
	1151.5	706.9	822.3	795.9
	1186.3	888.9	917.9	944.1
	1365.9	1134.3	1012.5	1055.4
	1403.1	1204.8	1189.7	1188.1
	1641.3	1221.6	1234.7	1264.9
	1659.6	1263.0	1317.9	1306.6
	1751.1	1308.9	1363.5	1312.8
	1773.1	1361.6	1417.2	1385.3
	1811.5	1395.7	1451.1	1404.8
	1869.9	1424.9	1530.5	1547.9
	1968.7	1426.3	1607.7	1574.9
	2011.4	1612.4	1633.9	1640.9
	2060.3	1656.9	1690.9	1689.9
	2118.1	1698.3	1743.0	1726.0
	2136.5	1748.6	1778.7	1762.4
	2226.5	1842.0	1785.8	1779.7
	2253.3	1853.0	1807.3	1829.0
RMSE	360.2	58.4	24.5	–
	N/A	–	[42.0]	–
MAD	53.7	38.3	23.7	–
	N/A	–	[25.5]	–

**Figure 9:**
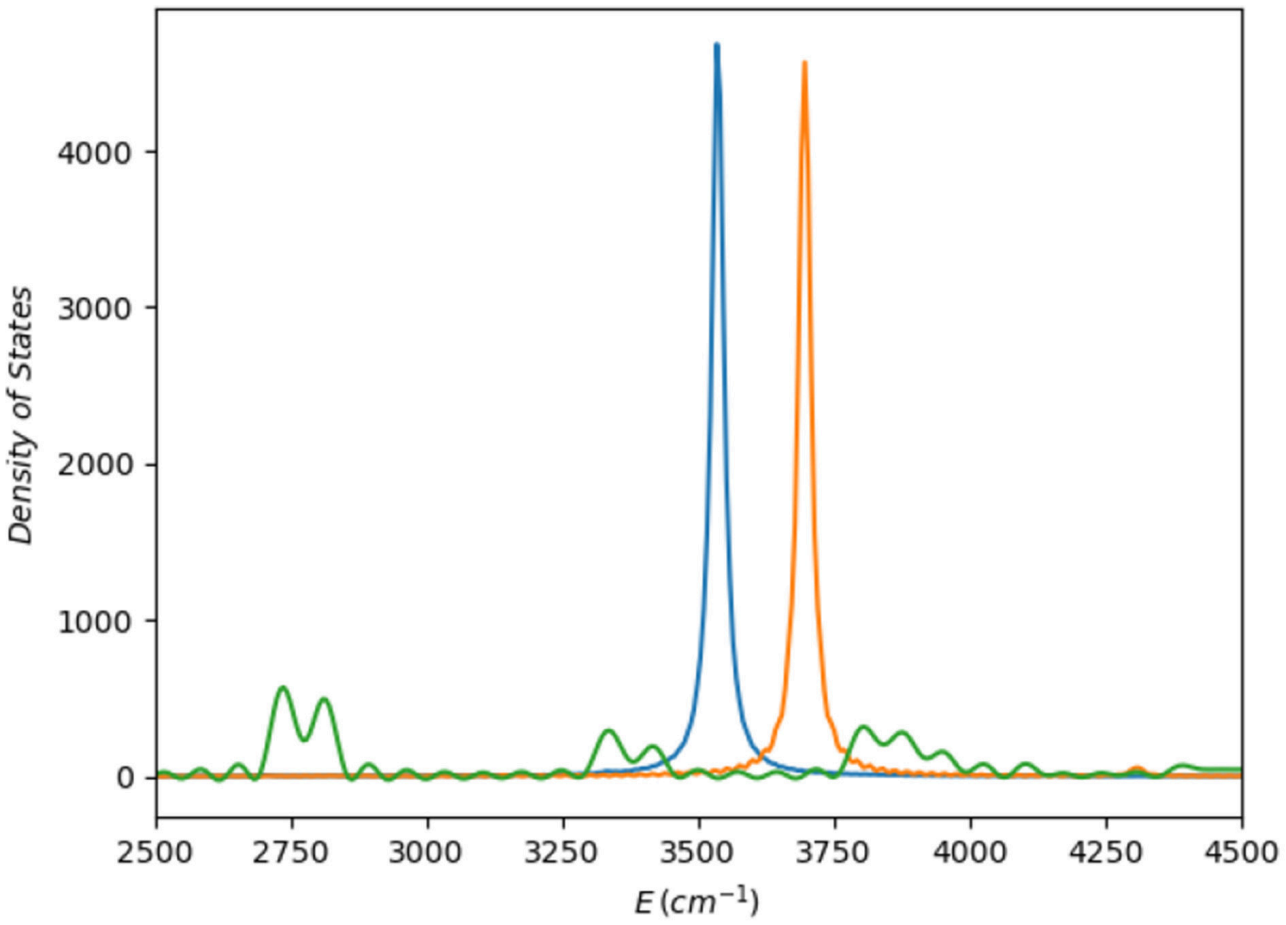
Vibrational spectra computed as the Fourier transform of the autocorrelation function obtained after excitation of one quantum in the OH stretching vibration centered the *cis* conformer region: (i) green line corresponds to the PM7-MGPF PES; (ii) orange line to the SRP-MGPF (**ζ**_1084_) PES; and (iii) blue line, the reference PES (*ab initio*) (Richter et al., 2004).

*Energies have been computed by MCTDH Block Improved Relaxation (see section 2.4). All PESs have been MGPFitted using a coarse grid consisting on 18,432 points, the so-called ev5 (see section 3.2.1). The first column presents the PM7-MGPF values (PM7), second and third correspond to SRP-MGPF with **ζ**_53_ and **ζ**_1084_, respectively. The last column presents the corresponding eigenenergies obtained using the analytical surface by Richter et al. (2004). The last four rows present the RMSE and the mean-absolute deviation (MAD) of each set of eigenvalues with respect to the analytical ones. The values in square brackets indicate the RMSE and MAD values taking into account the corresponding OH stretching anharmonic frequencies. The latter have been obtained through Fourier transform of an autocorrelation function (see Figure 9): (i) Analytical: 3533.8 cm^-1^ and (ii) **ζ**_1084_: 3695.7 cm^-1^. It should be noted that the PM7 values could not be determined (indicated by N/A) owing to a wrong behavior of the PM7-PES at this energy range (see Figure 9)*.

Finally, to take into account higher excited vibrational states, we have computed a vibrational spectrum by Fourier transform of the autocorrelation function corresponding to the dynamics of a wave packet generated by excitation of a quantum of energy in the OH stretching mode in the *cis* region of the potential. As observed (Figure 9), the PM7-MGPF spectrum is radically different to that of the reference PES, whereas the SRP-MGPF one shows the correct behavior. Apart from the, certainly not unexpected, shift in energy, both reference PES and SRP-MGPF reveal that the OH mode is practically uncoupled from the rest.

## 4. Conclusions and Future Prospects

We have introduced Specific Reaction Parameter Multigrid POTFIT (SRP-MGPF) a methodology which permits the generation of global chemically accurate Potential Energy Surfaces in sums-of-products (Tucker) form in a *quasi* black-box manner starting from a random input geometry. The SRP-MGPF workflow combines: (i) the automated determination of stationary points of a Potential Energy Surface (PES); (ii) the reparametrization of a Semiempirical Hamiltonian (SRP) using high-level *ab initio* data; and (iii) direct tensor-decomposition of the resulting SRP-PES with the Multigrid POTFIT (MGPF) algorithm. The resulting surface can be used with any on-the-fly dynamical software or, after MGPF, with grid-based quantum dynamical method, in particular the Multiconfiguration Time-Dependent Hartree (MCTDH) method. We have proven the validity of this method by fitting the SRP-MGPF PES for the HONO system in full dimensionality (6D) and reproducing, to a good agreement, the vibrational properties of a surface of CCSD(T)/cc-pVQZ quality. Current work deals with the extension of the method to treat coupled electronic excited states. To finalize, it should be highlighted that SRP-MGPF provides an inexpensive and accurate enough means of performing full-dimensional chemically meaningful quantum or classical simulations.

## Data Availability

All datasets generated for this study are included in the manuscript/Supplementary Files.

## Author Contributions

DP conceived the original idea, managed the project, and wrote the first draft of the manuscript. RP-B and DP were responsible for its implementation. RP-B was main responsible for the development of the necessary software. EM-N has been responsible for the TSSCDS calculations and classical trajectories. All authors contributed to manuscript revision, read and approved the submitted version.

### Conflict of Interest Statement

The authors declare that the research was conducted in the absence of any commercial or financial relationships that could be construed as a potential conflict of interest.
