# Supplementary material for: Specific Reaction Parameter Multigrid POTFIT (SRP-MGPF): Automatic Generation of Sum-of-Products Form Potential Energy Surfaces for Quantum Dynamical Calculations
Source: Front Chem. 2019 Aug 14;7:576. doi: 10.3389/fchem.2019.00576 (PMC6702682; doi:10.3389/fchem.2019.00576)
Supplement: Supplementary file 1 [file Data_Sheet_1.PDF]

Supplementary Material

1 LIST OF ACRONYMS

BBFS	Bond Breaking/Formation Search
BOBYQA	Bound Optimization BY Quadratic Approximation
DD-vMCG	Direct-Dynamics Variational Multiconfiguration Gaussian
DFT	Density Functional Theory
DOF	Degrees Of Freedom
DVR	Discrete Variable Representation
EOM	Equations Of Motion
G-MCTDH	Gaussian-Multiconfiguration Time-Dependent Hartree
HL	High Level
IR	Infrared
IRC	Intrinsic Reaction Coordinate
LIIC	Linear Interpolation in Internal Coordinates
LL	Low Level
MCTDH	Multiconfiguration Time-Dependent Hartree
MEP	Minimum Energy Path
MGPF	Multigrid POTFIT
MLSL	Multi-Level Single-Linkage
NN	Neural Network
PES	Potential Energy Surface
PM7	Parametric Method 7
QFF	Quartic Force Field
RMSE	Root-Mean Square Error
RXN	Reaction Network
SCF	Self-Consistent Field
SRP	Specific Reaction Parameters
TS	Transition State
TSSCDS	Transition State Search Using Chemical Dynamics Simulations
VCC	Vibrational Coupled Cluster
VCI	Vibrational Configuration Interaction
vdW-TSSCDS	van der Waals Transition State Search Using Chemical Dynamics Simulations
vMCG	Variational Multiconfiguration Gaussian
VPT2	Vibrational second-order Perturbation Theory
VSCF	Vibrational Self-Consistent Field
ZPE	Zero Point Energy

2 STAGES OF THE OPTIMIZATION PROCESS

In the present and next sections, we shall use roman numerals to refer to the different SRP optimization stages (see Table 1 in Section 3.1 in main text). The following correspondence between numeral and number of fitting points applies: I-53, II-367, III-546, IV-648, V-954, VI-1084.

Table S1. Value of the RMSE obtained in the global optimization process of the SRP parameters for the initial set of fitting points (*core*). The set is constituted by $N_p = 53$ geometries: the 3 stationary points of the system plus geometries taken from 1D and 2D full grids and randomly from a 6D grid. The optimization was carried out using the MLSL-BOBYQA algorithm combination and 100000 iterations.

N_p	RMSE (cm^{-1})
$3_{SP} + 26_{1D} + 14_{2D} + 10_{rnd6D} = 53$	806.84

Table S2. Values of the RMSE function for the *core* + 1D sets of N_p fitting points. Columns 2, 3 and 4 present: (i) the evaluation of the RMSE function using ζ_{53}, (ii) the RMSE after 2000 iteration BOBYQA using each set and (iii) the evaluation of the RMSE function using ζ_{1542}, respectively.

N_p	RMSE (cm^{-1}) / ζ_{53}	RMSE (cm^{-1}) / BOBYQA	RMSE (cm^{-1}) / ζ_{1542}
192	652.36	482.13	552.05
367	600.25	463.68	471.53
646	586.40	446.28	440.33
1029	579.89	413.06	424.72
1542	541.61	375.37	-
2088	528.76	365.13	369.97

Table S3. Values of the RMSE function for the *core* + $1D_{367}$ + 2D sets of fitting points. Columns 2, 3 and 4 present: (i) the evaluation of the RMSE function using ζ_{367}, (ii) the RMSE after 2000 iteration BOBYQA using each set and (iii) the evaluation of the RMSE function using ζ_{546}, respectively.

N_p	RMSE (cm^{-1}) / ζ_{367}	RMSE (cm^{-1}) / BOBYQA	RMSE (cm^{-1}) / ζ_{546}
546	442.03	437.26	-
1095	475.04	467.86	470.41
2027	479.44	468.05	474.99

Table S4. Values of the RMSE function for the *core* + $1D_{1542}$ + 2D sets of fitting points. Columns 2, 3 and 4 present: (i) the evaluation of the RMSE function using ζ_{1542}, (ii) the RMSE after 2000 iteration BOBYQA using each set and (iii) the evaluation of the RMSE function using ζ_{1721}, respectively.

N_p	RMSE (cm^{-1}) / ζ_{1542}	RMSE (cm^{-1}) / BOBYQA	RMSE (cm^{-1}) / ζ_{1721}
1721	375.26	366.88	-
2270	410.61	406.17	403.28
3202	433.42	426.48	426.71

Table S5. Values of the RMSE function for the *core* + 1D + 2D + $rnd6D$ sets of fitting points. Columns 2 and 3 present: (i) the evaluation of the RMSE function using ζ_{546}, (ii) the RMSE after 2000 iteration BOBYQA using each set.

N_p	RMSE (cm^{-1}) / ζ_{546}	RMSE (cm^{-1}) / BOBYQA
689	1095.29	701.30
1093	1529.18	871.40
1719	1716.59	948.30

Table S6. Values of the RMSE function for the *core* + 1D + 2D + $rnd6D_{SP}$ sets of fitting points. The subindex SP indicates that we restrict our random points to the vicinity of the stationary points. Columns 2 and 3 present: (i) the evaluation of the RMSE function using ζ_{546}, (ii) the RMSE after 2000 iteration BOBYQA using each set.

N_p	RMSE (cm^{-1}) / ζ_{546}	RMSE (cm^{-1}) / BOBYQA
648	449.62	442.47
849	449.25	419.08
1148	446.99	406.35

Table S7. Values of the RMSE function for the *core* + 1D + 2D + $rnd6D_{SP}$ + LIIC-IRC set of fitting points. Columns 2, 3 and 4 present: (i) the evaluation of the RMSE function using ζ_{648}, (ii) the RMSE after 2000 iteration BOBYQA using each set and (iii) the corresponding MLSL-BOBYQA optimization of the set with 10000 iterations, respectively.

N_p	RMSE (cm^{-1}) / ζ_{648}	RMSE (cm^{-1}) / BOBYQA	RMSE (cm^{-1}) / MLSL-BOBYQA
945	386.34	382.15	382.15

Table S8. Value of the RMSE function for the *core* + 1D + 2D + $rnd6D_{SP}$ + LIIC-IRC + rnd(LIIC) set of fitting points (energies bellow 5000 cm^{-1}). Column 2 contains the corresponding BOBYQA optimizations of the set with 2000 iterations.

N_p ($E < 5000\,cm^{-1}$)	RMSE (cm^{-1}) / BOBYQA
1084	331.82

Table S9. Value of the RMSE function for the validation set of fitting points (energies bellow 12000 cm^{-1}). Column 2 contains the evaluation of the RMSE function using ζ_{1084}.

N_p ($E < 12000\,cm^{-1}$)	RMSE (cm^{-1}) / ζ_{1084}
1200	355.88

3 GEOMETRIES OF THE STATIONARY POINTS

Table S10. Geometry of the transition state structure at the CCSD(T)/cc-pVQZ *ab initio* level of theory and corresponding semiempirical values for the PM7 method and the SRPs in the different stages of the optimization.

Transition State						
	r_{NO} (Å)	r_{OH} (Å)	$r_{N=O}$ (Å)	θ_1 (°)	θ_2 (°)	ρ (°)
Ab initio	1.507	0.962	1.165	100.700	110.500	86.400
PM7	1.401	0.985	1.167	109.44	114.53	90.491
I	1.580	0.981	1.159	98.850	111.395	80.862
II	1.560	0.986	1.166	98.095	110.907	80.124
III	1.554	0.985	1.167	98.523	111.101	80.435
IV	1.551	0.981	1.166	98.684	111.110	80.435
V	1.549	0.980	1.168	98.888	111.028	81.042
VI	1.515	0.969	1.167	98.930	110.318	83.697

Table S11. Geometry of the *cis* structure at the CCSD(T)/cc-pVQZ *ab initio* level of theory and corresponding semiempirical values for the PM7 method and the SRPs in the different stages of the optimization.

CIS						
	r_{NO} (Å)	r_{OH} (Å)	$r_{N=O}$ (Å)	θ_1 (°)	θ_2 (°)	ρ (°)
Ab initio	1.392	0.975	1.184	104.400	113.200	0.000
PM7	1.339	1.003	1.178	115.320	117.995	0.001
I	1.391	0.998	1.176	108.371	115.186	0.001
II	1.384	0.999	1.179	108.128	113.802	-0.001
III	1.381	0.999	1.179	108.271	113.988	0.002
IV	1.382	0.995	1.178	108.380	113.976	0.002
V	1.382	0.994	1.178	108.423	113.964	0.001
VI	1.397	0.985	1.181	106.602	114.065	0.000

Table S12. Geometry of the *trans* structure at the CCSD(T)/cc-pVQZ *ab initio* level of theory and corresponding semiempirical values for the PM7 method and the SRPs in the different stages of the optimization.

TRANS						
	r_{NO} (Å)	r_{OH} (Å)	$r_{N=O}$ (Å)	θ_1 (°)	θ_2 (°)	ρ (°)
Ab initio	1.427	0.965	1.171	101.900	110.700	180.000
PM7	1.384	0.997	1.172	105.908	112.397	179.999
I	1.489	0.978	1.167	99.711	110.629	179.996
II	1.453	0.977	1.172	99.326	110.487	-179.998
III	1.450	0.976	1.172	99.667	110.636	-179.999
IV	1.448	0.973	1.172	99.697	110.676	-180.000
V	1.446	0.972	1.172	100.184	110.551	180.000
VI	1.440	0.964	1.176	99.530	109.222	-179.999

4 VALUES OF THE SEMIEMPIRICAL PARAMETERS

Table S13. Specific reaction parameters values for the different stages of the optimization process. Labels follow the openMOPAC nomenclature.

Specific Reaction Parameters							
	Atom	I	II	III	IV	V	VI
USS	H	-8.172	-8.007	-7.973	-7.964	-8.076	-8.096
BETAS	H	-8.384	-8.351	-8.336	-8.404	-8.399	-8.374
ZS	H	0.882	0.863	0.863	0.863	0.862	1.010
GSS	H	18.390	17.824	17.826	17.687	17.693	17.584
USS	N	-51.226	-52.708	-52.728	-52.726	-52.778	-53.444
UPP	N	-34.444	-34.526	-34.545	-34.513	-34.531	-35.902
BETAS	N	-26.451	-25.713	-25.813	-25.808	-25.789	-21.257
BETAP	N	-19.536	-19.428	-19.372	-19.367	-19.363	-15.940
ZS	N	1.692	1.677	1.674	1.687	1.688	1.454
ZP	N	1.631	1.599	1.601	1.603	1.600	1.639
GSS	N	8.329	8.225	8.226	8.228	8.224	8.241
GSP	N	7.573	7.563	7.565	7.569	7.565	8.634
GPP	N	15.873	16.289	16.302	16.259	16.248	16.269
GP2	N	7.210	7.255	7.249	7.257	7.269	7.563
HSP	N	2.084	2.029	2.022	2.023	1.997	1.686
USS	O	-103.603	-103.943	-103.987	-103.909	-103.987	-110.152
UPP	O	-72.157	-72.259	-72.351	-72.408	-72.375	-71.064
BETAS	O	-80.241	-79.835	-79.761	-79.015	-79.031	-81.147
BETAP	O	-26.306	-26.155	-26.304	-26.307	-26.303	-22.463
ZS	O	4.225	4.243	4.236	4.224	4.224	4.196
ZP	O	2.258	2.325	2.321	2.327	2.328	2.258
GSS	O	19.441	19.355	19.405	19.426	19.425	20.504
GSP	O	17.694	18.178	18.177	18.175	18.170	16.588
GPP	O	15.335	15.172	15.179	15.186	15.168	15.056
GP2	O	12.831	12.825	12.846	12.858	12.845	12.553
HSP	O	3.691	3.716	3.700	3.649	3.571	2.404
ALPB_N	H	1.365	1.728	1.725	1.729	1.750	1.542
XFAC_N	H	0.140	0.141	0.142	0.121	0.148	0.148
ALPB_O	H	1.774	1.733	1.735	1.738	1.741	1.823
XFAC_O	H	0.209	0.205	0.205	0.204	0.204	0.208
ALPB_O	N	1.989	2.009	2.008	2.011	2.012	2.187
XFAC_O	N	0.594	0.597	0.596	0.596	0.597	0.596
ALPB_O	O	2.624	2.622	2.620	2.618	2.618	2.589
XFAC_O	O	0.289	0.298	0.299	0.302	0.299	0.299

5 COMPARISON OF EXACT (TUCKER FORM) EXPANSION AND MGPF TENSOR DECOMPOSITION

Table S14. Comparison of the computational details of each type of MGPF tensor decomposition with an exact (Tucker form) expansion. The primitive (fine) grid is composed of a total $2.804 \cdot 10^7$ points. Coarse grids have been generated by (automatically) selecting *every* n-th (n=3, 4, 5) fine grid point for each individual DOF. The resulting coarse grid points consist on 172800 ($ev3$), 51200 ($ev4$), or 18432 ($ev5$) coarse grid points.

PES	Coarse grid	RMSE (cm^{-1})	CPU time (min)	Size (MB)
Exact	-	0.0	234.2	214.0
MGPF	$ev3$	0.8	22.0	1.4
	$ev4$	21.0	8.3	0.4
	$ev5$	117.8	3.6	0.2

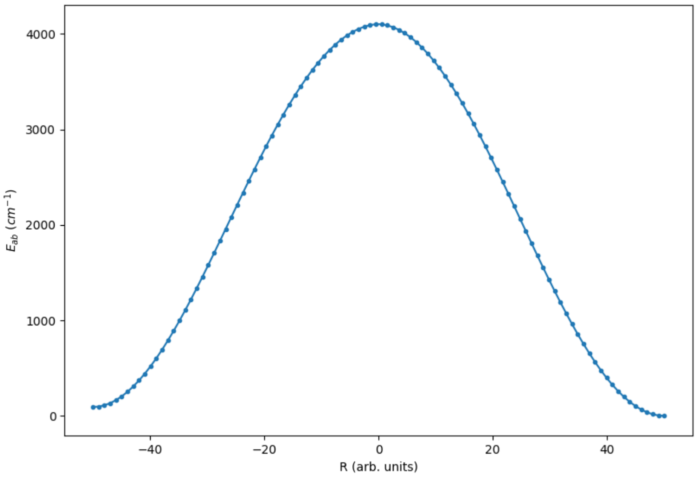

Figure S1. Energies of the geometries obtained through a two-piece linear interpolation in internal coordinates (LIIC) between the: (i) cis-conformer (MIN1, negative end of the x-axis) and the TS (TS1, x=0); and (ii) the TS (TS1, x=0) and the trans-conformer (MIN2, positive end of the x-axis).
